# Data on microRNAs and microRNA-targeted mRNAs in *Xenopus* ectoderm

**DOI:** 10.1016/j.dib.2016.09.054

**Published:** 2016-10-14

**Authors:** Vrutant V. Shah, Benjamin Soibam, Ruth A. Ritter, Ashley Benham, Jamina Oomen, Amy K. Sater

**Affiliations:** a University of Houston, Houston, TX, USA; b Texas Heart Institute, Houston, TX, USA; c Stonybrook Univ., Stonybrook, NY, USA; d Department of Computer Science and Engineering technology, University of Houston-Downtown, Houston, TX, USA

**Keywords:** microRNA, Neural, Ectoderm, *Xenopus*

## Abstract

Small RNAs from early neural (i.e., Noggin-expressing, or NOG) and epidermal (expressing a constitutively active BMP4 receptor, CABR) ectoderm in *Xenopus laevis* were sequenced to identify microRNAs (miRs) expressed in each tissue. Argonaute-associated mRNAs were isolated and sequenced to identify genes that are regulated by microRNAs in these tissues. Interactions between these ectodermal miRs and selected miR-regulated mRNAs were predicted using the PITA algorithm; PITA predictions for over 600 mRNAs are presented. All sequencing data are available at NCBI (NCBI Bioproject Accession number: PRJNA325834). This article accompanies the manuscript “MicroRNAs and ectodermal specification I. Identification of miRs and miR-targeted mRNAs in early anterior neural and epidermal ectoderm” (V.V. Shah, B. Soibam, R.A. Ritter, A. Benham, J. Oomen, A.K. Sater, 2016) [1].

**Specifications Table**

**Table:** 

Subject area	Developmental Biology and Genomics
More specific subject area	microRNAs and early ectodermal development
Type of data	Supplementary tables
How data was acquired	Next-Generation Sequencing; multiple instruments
Data format	Analyzed
Experimental Factors	Xenopus ectoderm in which BMP signals are either inhibited (NOG) or activated (CABR) to give rise to either neural or epidermal tissue, respectively.
Experimental features	We generated ectoderm overexpressing either noggin (NOG) to elicit an anterior neural state, or a constitutively active BMP4 receptor (CABR) to elicit an epidermal state of specification. These tissues were used to prepare microRNAs and argonaute-associated mRNAs (thus regulated by microRNAs) for sequencing and analysis.
Data source location	Houston, TX, USA
Data accessibility	Data are submitted with this publication; sequencing reads are also available through NCBI Bioproject Accession number: PRJNA325834 at http://www.ncbi.nlm.nih.gov/bioproject/?term=PRJNA325834

**Value of the data**

• Sequence data and target predictions provide a foundation for subsequent functional analyses of miR-mRNA interactions. Large-scale microRNA target predictions have not previously been generated for *Xenopus laevis*.
• These datasets can support future studies on microRNA-dependent translational control in embryonic systems, and they can be used to establish the extent of conservation of microRNA-targeted mRNA interactions.
• These datasets can be used to investigate the roles of microRNAs in the establishment of neural vs epidermal ectoderm, the transition from the early neural gene regulatory network to the neural proliferative and neurogenic networks, and the restriction of pluripotency in embryonic ectoderm.

## 1 Data

These data include:

1) The identification and genomic locations of microRNAs expressed in early neural and epidermal ectoderm from *Xenopus laevis* embryos. Sequence reads for 3 biological replicates, as well as the accompanying DESeq analysis, are provided. (Supplementary Table 1 in Ref. [1] and Supplementary Table 2).
2) The identification of RNAs in the Argonaute Ribonucleoprotein complex (Ago–RNP) from in early neural and epidermal ectoderm from *Xenopus laevis* embryos (Supplementary Table 3). Total RNAs present in both samples are also identified (Supplementary Table 4).
3) Predicted miR-mRNA interactions for “High Confidence” miR-targeted ectodermal mRNAs from the Ago–RNP pools for early neural and epidermal ectoderm (Supplementary Table 5).
4) Gene Ontology (GO) categories and associated genes among the “High Confidence” miR-targeted ectodermal mRNAs (Supplementary Table 6).
5) Conserved targets of pou5f3 among the miR-targeted mRNAs for NOG and CABR Ago-associated mRNAs and predictions of miR –mRNA interactions for the genes (Supplementary Table 7).

## 2 Experimental design, materials and methods

For microRNAs: Small RNA sequencing was carried out on paired NOG and CABR ectodermal samples in 3 biological replicates.

For Ago-RNP RNAs: RNA sequencing was carried out on RNAs immunoprecipitated from paired NOG and CABR ectodermal samples in 3 biological replicates.

### 2.1 Methods

#### 2.1.1 Preparation of embryonic tissue samples

Detailed methods for the preparation of embryonic tissue samples are presented in Shah et al. [1].

#### 2.1.2 Preparation and sequencing of small RNA libraries

After lysing the midgastrula animal caps in Trizol, the Direct-zol RNA mini-prep kit (Zymo) was used to purify the RNA. Libraries were generated from these DNAseI-treated RNA samples using the NEBNext Multiplex Small RNA Library Prep Set for Illumina kit. Library yields were quantified using the Quant-iT^™^ Picogreen dsDNA reagent (Thermofisher). Sequencing of these libraries was carried out at the M. D. Anderson Sequencing and Microarray Facility.

#### 2.1.3 Co-immunoprecipitation of Ago–RNP complexes and isolation of associated RNA

Detailed methods for co-immunoprecipitation of Ago–RNP complexes and isolation of RNP-associated RNA are presented in Shah et al. [1]; methods are modified from [4].

#### 2.1.4 Preparation and sequencing of Ago–RNP RNA libraries

RNA isolated from the immunoprecipitated Ago–RNP samples was used to generate sequencing libraries via the ScriptSeq^™^ Complete Gold Kit – Low Input (Epicentre Technologies); these samples were not subjected to rRNA depletion before library preparation. Sequencing was carried out at the M. D. Anderson Cancer Center Sequencing and Microarray Facility.

#### 2.1.5 Preparation and sequencing of total RNA libraries

The Ribo-ZeroTM rRNA kit was used to deplete the total RNA or “input RNA”) samples of ribosomal RNA, prior to subsequent purification using the RNeasy MinElute Cleanup Kit. Sequencing libraries were then generated using the ScriptSeq^™^ Complete Kit – Low Input from Epicentre (Cat.No. SCL6H). Sequencing was carried out by the Sequencing Core Facility at the University of Houston. Sequence reads for all sequencing studies reported here are publicly available through NCBI (NCBI Bioproject Accession number: PRJNA325834).

#### 2.1.6 Analysis of sequencing data

A) ***Mapping of small RNA sequence reads***
  Raw sequence reads for small RNAs were evaluated using FastQC (v0.11.2) and trimmed with Cutadapt (options: −a for 3′ adapter, −g for 5′ adapter, minimum retention length 17 bp). The *Xenopus laevis* genome assembly 9.1 served as the basis for the genome reference index, which was constructed using Bowtie 1.1.1 (Langmead et al. [6], option bowtie-build). miRDeep v.2 Friedländer et al. [3] was used to align mapped sequences with the genome reference index, with additional processing via the mapper.pl script (options: −e for input file in fastq format, −p for reference genome and −t for printing read mappings to.arf file). A miRDeep2.pl script was used to carry out a second alignment of all aligned reads to the miRBase 21 human and *Xenopus tropicalis* miR datasets. As in our previous study [6], we used Bedtools to identify 75 bp sequences flanking the putative miR sequences in the *X. laevis* genome; these candidate precursor-miR sequences were then assessed for characteristic stem-loop secondary structure using RNAfold Lorenz et al. [7].
B) ***Analysis of small RNA expression***
  A non-redundant set of miRs was generated by comparisons of reads aligned to human vs *X. tropicalis* miR datasets. We used DeSeq Anders and Huber [2] to identify relative levels of expression for specific miRs in neural ectoderm vs epidermal ectoderm. Differential expression of individual miRs was based on a negative binomial distribution, which yielded normalized values relative to the total miR reads as well as identifying miRs that are candidates for differential expression. The results are presented as follows: (1) miR IDs (2) average read counts (3) average read counts from epidermal ectoderm (4) average read counts from neural ectoderm (5) the -fold difference between neural and epidermal expression (6) the log2 values of fold difference (neural miRs/epidermal miRs) (7) *P* values.
C) ***Mapping and analysis of sequences from Ago–RNP RNA and Total RNA samples***
  Libraries prepared from the ago–RNA samples were subjected to paired-end sequencing. Cutadapt was used to trim adapter sequences using the following options: −b for both 3′ and 5′ adapter trimming because of the variable sequence length. The reference index was prepared from *Xenopus laevis* transcriptome dataset (courtesy of Taejoon Kwan and Ed Marcotte, UT Austin). Bowtie2 was used to align paired-end sequences to the transcriptome index using the following options: --local for local alignments, −p 8 to run parallel 8 search threads and −S for output in sam format). Sam output files were converted to Bam files using Samtools; express Roberts and Pachter [8] was used to establish FPKM values and generate annotations for the aligned reads.
D) ***Identification of differentially represented Ago–mRNAs***
  We omitted all transcripts with representation below threshold (<5 read counts) in all libraries. DESeq was used to normalize the levels of specific Ago–RNAs for non-specific binding, and to determine which transcripts showed a difference in representation between neural and epidermal tissues. Transcripts were normalized individually to total numbers of transcripts in each library. DESeq was used to determine the ratio of log2FoldChange (log2FC) in neural/epidermal ectoderm for each transcript using a negative binomial distribution. The thresholds for significant differential representation were (1) a log2FC of >2 and (2) a *p* value of <0.01.
E) ***Sequence analysis of total RNAs***
  Bowtie2 was used to align reads from total RNA samples against the transcriptome-based index, using the following options:.−q for fastq input file format, --sensitive-local as a default mode in local alignments, −I 200 and −X 300 to set minimum and maximum fragment length valid for paired end alignments, −p 12 to run 12 parallel search threads. The resulting output was evaluated with eXpress to annotate transcripts represented in the “total RNA” pools for both neural and epidermal samples, yielding annotations, read counts, and FPKM values.

#### 2.1.7 Computational predictions of miR-mRNA interactions

The PITA algorithm (Probability of Interaction by Target Site Accessibility (PITA), Kertesz et al. [5]) was used to generate “high-confidence” predictions of miR-mRNA interactions.

## Appendix A Supplementary material

**Supplementary Table 2. Identification of miRs from NOG and CABR ectoderm**. The first worksheet lists all miRs identified by alignment to either hsa-miRs or xtr-miRs from miRBase 21, with genomic locations and mature miR sequences. The second sheet shows the read counts in each sample for all miRs. The third sheet provides the DESeq output data (base means, *p* values, log2 fold change, etc.) for all miRs.

**Supplementary Table 3. Epidermal vs neural Ago–RNP RNAs**. The first sheet provides the DESeq analysis of the Ago–RNP RNAs. The second sheet includes read counts for all biological replicates.

**Supplementary Table 4. Analysis of Total RNAs from NOG and CABR ectoderm**. The first sheet contains the DESeq analysis for the ribo-depleted total RNAs. The second sheet includes read counts for all biological replicates.

**Supplementary Table 5. High Confidence Ago–RNP RNAs and associated PITA predictions for miR–mRNA interactions**. The first sheet lists the High Confidence (“HC”) genes from the NOG Ago–RNP RNAs, while the second sheet provides PITA predictions for interaction of ectodermal miRs with the HC NOG genes, including both High Probability (DDG≤−10) and moderate probability (DDG≤−5) predictions for all genes for which sufficient 3′UTR sequence is available. The PITA output includes the gene ID, the miR, the start and end locations of the seed sequence in the 3′UTR, the base pairing across the seed sequence, DG values for the duplex and open conformations at the site of the seed sequence, and the difference in DG between the duplex and open conformations (DDG). The third and fourth sheets provide the same information for the HC CABR genes and PITA predictions.

**Supplementary Table 6. Partial Gene Ontology Analysis for HC NOG and HC CABR RNAs**. The two sheets list selected Gene Ontology categories, p values, and associated genes for the HC NOG and HC CABR gene sets, respectively.

**Supplementary Table 7. Conserved*****pou5f3*****targets represented in the ectodermal Ago-RNA pools and associated PITA predictions for miR-mRNA interaction**. The first sheet lists the conserved oct4 targets, as identified by Livigni et al. (2013), that are present in the ectodermal Ago–RNA pools, and the corresponding genes. The second sheet provides PITA predictions for interaction of ectodermal miRs with the *pou5f3* target genes, including both High Probability (DDG≤−10) and moderate probability (DDG≤−5) predictions for all genes for which sufficient 3′UTR sequence is available. The PITA output includes the gene ID, the miR, the start and end locations of the seed sequence in the 3′UTR, the base pairing across the seed sequence, DG values for the duplex and open conformations at the site of the seed sequence, and the difference in DG between the duplex and open conformations (DDG).
